# 

**Published:** 1864-04

**Authors:** 


*The Army Surgeon.* By General Baron AMBERT.

*Contents.*

ORIGINAL COMMUNICATIONS........................................ 49
1. On the Microscopic Anatomy, Physiology and Pathology of the Human Liver, by Surgeon H. D. Schmidt. 2. New Test Paper, suggested by Private J. C. Wharton, of Atlanta, Georgia. 3. New Method of Treating Ununited Fracture of Long Bones, by Surgeon James Bolton. 4. Resection of Upper-half of Humerus, reported by Surgeon John Stainback Wilson. 5. Chronic Aphonia cured during an attack of Pneumonia, by Assistant-Surgeon Thomas Kinchley.

CONFEDERATE STATES HOSPITAL REPORTS.................. 57
Eleven Cases of Compound Fracture of Cranium by Gun-shot Wound treated at Chimborazo Hospital.

EDITORIAL AND MISCELLANEOUS................................ 59
Artificial Limbs—How to Make Them. Obituary.

TRANSACTIONS OF THE ASSOCIATION OF ARMY AND NAVY SURGEONS................................................ 60

CHRONICLE OF MEDICAL SCIENCE................................ 62

The Causes of Hysteria, by Professor Brown-Sequard and Professor Jacksh, of Prague. Remarks on the Recently Proposed American Plan of Treating Gun-shot Wounds of the Chest by "Hermetically Sealing," by Deputy Inspector-General T. Longmore, Professor of Military Surgery. Southern Surgeons Abroad. Recto-Vesical Lithotomy. The Army Surgeon, by General Baron Ambert. Baron Larrey. M. Velpeau. Ovariotomy.

